# Improving dialogue among researchers, local and indigenous peoples and decision-makers to address issues of climate change in the North

**DOI:** 10.1007/s13280-019-01277-9

**Published:** 2019-11-12

**Authors:** Terry V. Callaghan, Olga Kulikova, Lidia Rakhmanova, Elmer Topp-Jørgensen, Niklas Labba, Lars-Anders Kuhmanen, Sergey Kirpotin, Olga Shaduyko, Henry Burgess, Arja Rautio, Ruth S. Hindshaw, Leonid L. Golubyatnikov, Gareth J. Marshall, Andrey Lobanov, Andrey Soromotin, Alexander Sokolov, Natalia Sokolova, Praskovia Filant, Margareta Johansson

**Affiliations:** 1 University of Sheffield, Alfred Denny Building, Western Bank, Sheffield, S10 2TN UK; 2 28 Promyshlennaya str, Saint-Petersburg, Russia 190121; 3 Tomsk State University, 36 Lenina Pr, Tomsk, Russia 634050; 4 University of Konstanz, Constance, Germany; 5 Department of Bioscience, Arctic Research Center, Aarhus University, Frederiksborgvej 399, Building 7418, I2.41, 4000 Roskilde, Denmark; 6 Centre for Sámi Studies, University of Tromsø, Postboks 6050, Langnes, 9037 Tromsö, Norway; 7 Gabna Sámi Village, Scandinavia, Norway; 8 British Antarctic Survey, UK Natural Environment Research Council Arctic Office, High Cross, Madingley Road, Cambridge, CB3 0ET UK; 9 Thule Institute, University of Oulu and University of the Arctic, P.O. Box 7300 90014, Oulu, Finland; 10 Norconsult AS, Kjørboveien 22, 1338 Sandvika, Norway; 11 A.M. Obukhov Institute of Atmospheric Physics, 3 Pyzhevsky Lane, Moscow, Russia 119017; 12 Arctic Research Centre of the Yamal-Nenets Autonomous District, Line 8, Nadym, Russia 629730; 13 Research Institute of Ecology and Natural Resources Management, Tumen State University, 6 Volodarskogo St, Tyumen, Russia 625003; 14 Arctic Research Station, Institute of Plant & Animal Ecology Ural Branch, Russian Academy of Sciences, 21, Str. Zelenaya Gorka, Labytnangi, Russia 629400; 15 Reindeer Herders Association of the Yamal-Nenets Autonomous District, of. 35, 41 Sverdlov Str, Salekhard, Russia 629007; 16 Department of Physical Geography and Ecosystem Science, Lund University, Solvegatan 12, 223 62 Lund, Sweden; 17 Institute of the Biological Problems of the North, Russian Academy of Sciences, Portovaya Street 18, Magadan, Russia 685000; 18 Arctic Research Center of Yamal-Nenets Autonomous District, 73, Str. Respublika, Salekhard, Russia 629008

**Keywords:** Dialogue, Environmental change, Indigenous peoples, Policy-makers, Researchers, Siberia

## Abstract

The Circumpolar North has been changing rapidly within the last decades, and the socioeconomic systems of the Eurasian Arctic and Siberia in particular have displayed the most dramatic changes. Here, anthropogenic drivers of environmental change such as migration and industrialization are added to climate-induced changes in the natural environment such as permafrost thawing and increased frequency of extreme events. Understanding and adapting to both types of changes are important to local and indigenous peoples in the Arctic and for the wider global community due to transboundary connectivity. As local and indigenous peoples, decision-makers and scientists perceive changes and impacts differently and often fail to communicate efficiently to respond to changes adequately, we convened a meeting of the three groups in Salekhard in 2017. The outcomes of the meeting include perceptions of how the three groups each perceive the main issues affecting health and well-being and recommendations for working together better.

**Electronic supplementary material:**

The online version of this article (doi:10.1007/s13280-019-01277-9) contains supplementary material, which is available to authorized users.

## Introduction

**Fig. 1:**
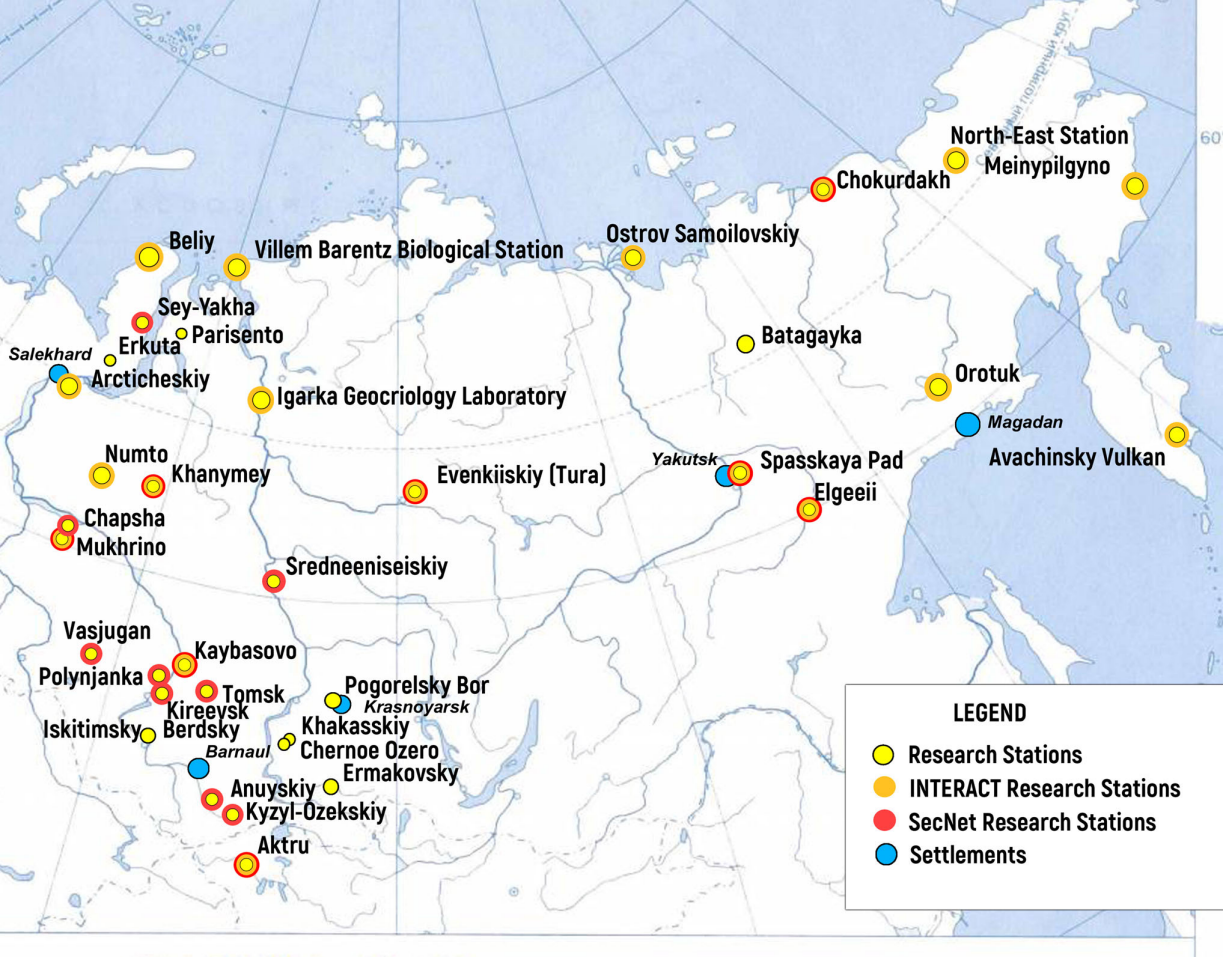
Research stations in Siberia

The Circumpolar North has been changing rapidly within the last decades, and the socioeconomic systems of the Eurasian Arctic and Siberia in particular have displayed the most dramatic changes (Forbes et al. 2009; Kumpula et al. 2012). In this region, anthropogenic drivers of environmental change such as migration, industrialization and urbanization (Orttung and Reisser 2014) are added to climate-induced changes in the natural environment, for example warming of the atmosphere, reduced area of sea ice, permafrost thawing and increased frequency of extreme events (AMAP 2017a). Understanding and adapting to both types of changes are important to both the local peoples in the Circumpolar North (Anisimov and Orttung 2018) and the wider global community as changes to climate conditions in one part of the Earth have knock-on effects for other regions (e.g. Francis and Vavrus 2012). Coping with threats and gaining benefits from the evolving changes are both possible responses but choices should be informed by the best available knowledge. Global warming has been most pronounced in the Arctic during the last decades (Overland et al. 2017). The largest landmass in the region, Siberia (total area 13 million km^2^; Fig. 1), is, however, relatively little studied. Because of the vast size of Siberia, its impacts on its local populations and its potential feedbacks to global climate, it should be a priority for research. Nevertheless, Siberia should not be studied in isolation: it is part of the Earth System and several environmental and socioeconomic processes share analogues in other Arctic regions, e.g. permafrost thaw, migration and traditional life style changes. Studies which compare processes and responses between Arctic regions are therefore crucial in order to expedite dissemination of knowledge and coping strategies.

**Fig. 2:**
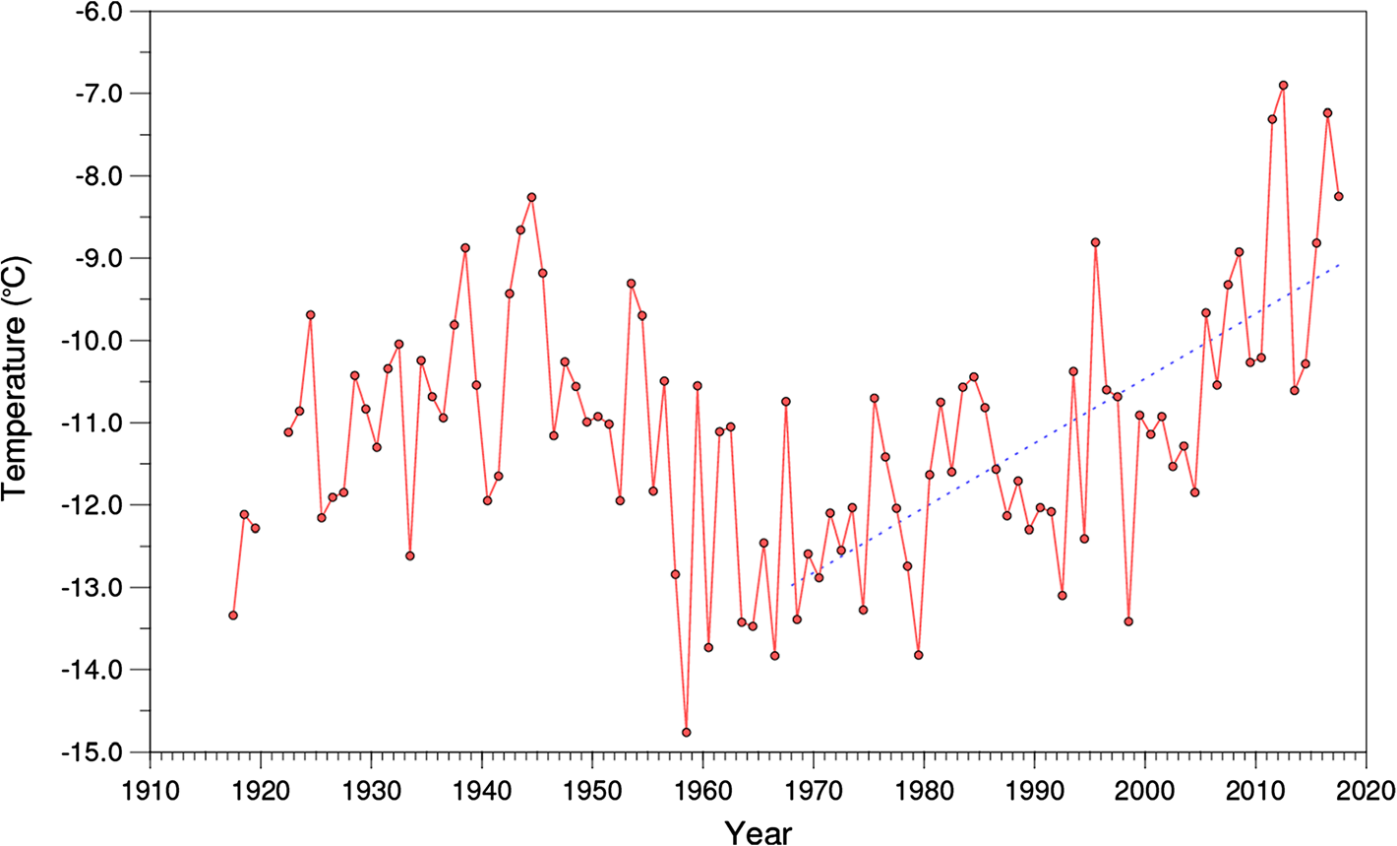
Mean annual temperature record from Ostrov Dickson station (73.5° N, 80.3° E). The dashed line shows the ~ 4 °C warming in the past 50 years (1968–2017). Data were acquired from the Russian Research Institute of Hydrometeorological Information World Data Centre (RIHMI-WDC) (http://aisori.meteo.ru/ClimateE) with some missing values infilled from the Koninkklijk Nederlands Meteorologisch Institute (KNMI) Climate Explorer data portal (https://climexp.knmi.nl) and the Berkeley Earth dataset (http://berkelyearthlbl.gov/stations/169952)

Attempts to cap the global temperature increase by mitigation of CO_2_ emissions have so far failed. Compared to the initial caps set in the Paris Agreement (Horowitz 2016), of 1 °C, and then below 2 °C globally, compared to pre-industrial levels, the mean annual surface temperature in the north of Siberia has increased markedly by about 4 °C over the past 50 years (Fig. 2). Also, the frequency and intensity of weather extremes (both rainfall and temperature) have increased with an associated increase in the frequency of large and severe wildfires and dust storms which have impacts that often extend far beyond Siberia and Northern Eurasia, affecting even global markets and raising concerns about global food security (Groisman et al. 2017).

Therefore, the need to adapt to a future, warmer climate in Siberia is urgent. Simplistically, adaptation requires (i) identification of the problem by researchers and local peoples, (ii) planned responses by researchers, local peoples and decision-makers, (iii) implementation of planned responses by decision-makers and local peoples and (iv) monitoring of the effects of adaptation and mitigation strategies by researchers and local peoples. This scheme requires that researchers, local peoples and decision-makers work together better than at present. To achieve this goal and to provoke thoughtful dialogues, an international meeting was held to bring together representatives of local and indigenous peoples, researchers and decision-makers, with a focus on issues related to climate change impacts in the Arctic.

The methodology behind this study is to summarize the results of the 2nd Siberian Environmental Change Network (SecNet: http://www.secnet.online/home-eng.html) workshop “Winter Weather and Climate Extremes: how can researchers, authorities and local peoples work together to record, predict and adapt?” held in Salekhard, Russia on 1–3rd November 2017. The workshop was co-organized by INTERACT (https://eu-interact.org/). The workshop’s methodology was to bring representatives of the three groups (researchers, local peoples and decision-makers) together. The main objective of this study was to explore how to improve communications, make research more relevant to local needs and facilitate adaptation actions. The methodology and format of the meeting gave the participants an opportunity to discuss issues of concern to them in groups and to present the main outcomes of the discussion in plenum to identify possible solutions for future collaboration. In this study, we present issues perceived as important by each group and present jointly agreed recommendations.

## Issues perceived by researchers

### The main issues affecting the Siberian environment and ecosystems

Increasing temperatures and changes in precipitation patterns which impact the wider Siberian environment (AMAP 2017a, b) are caused by an increase in global carbon emissions (from industry, heating, transport, farming, etc.) (Bindoff et al. 2013). However, anthropogenic activities such as mining, the oil industry, waste disposal, grazing, fishing, hunting and logging often have greater local impacts.

**Fig. 3:**
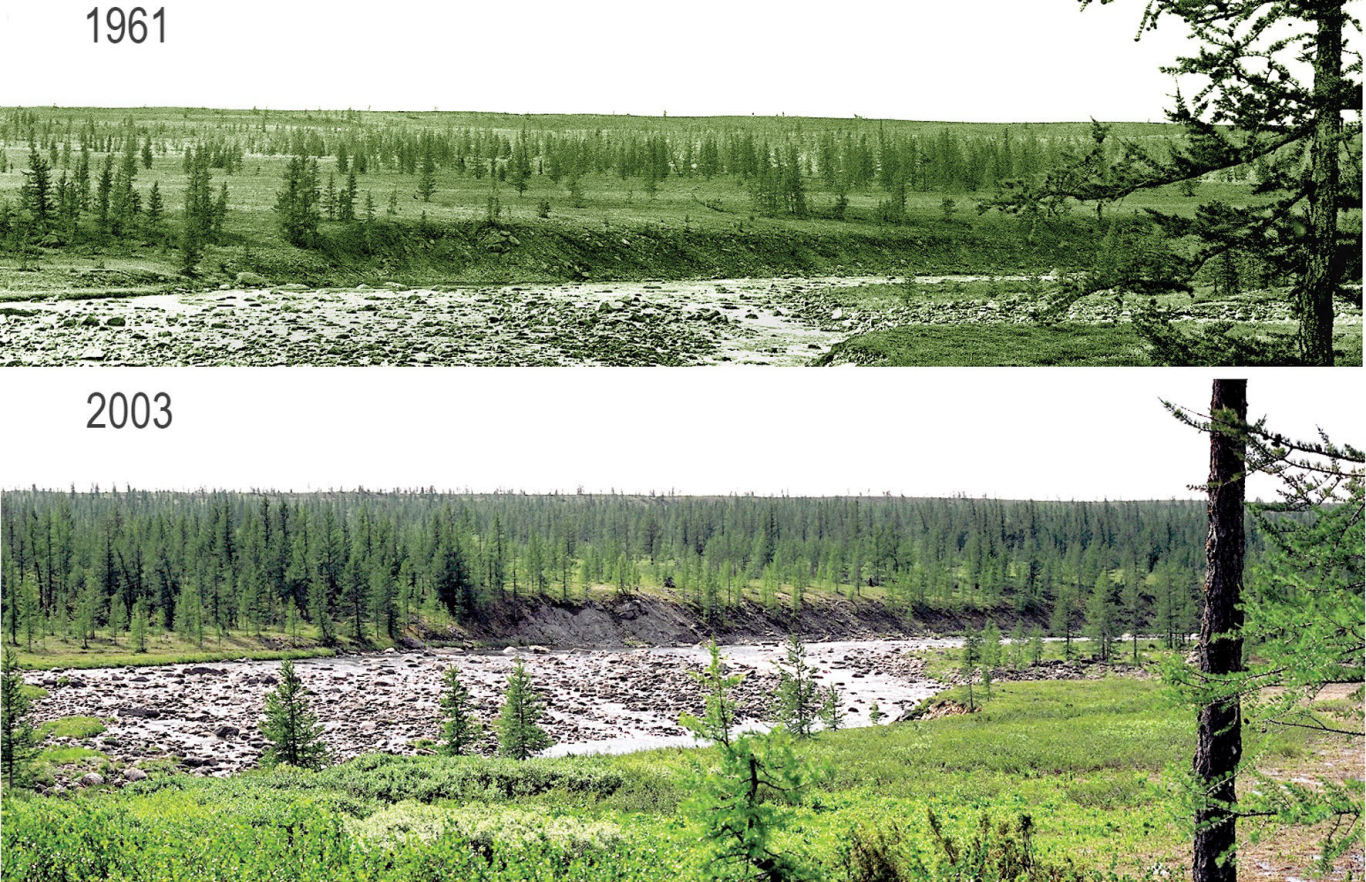
Thickening of the larch forest on the left banks of the Enga-Yu River, Polar Urals, near Salekhard (Photos: Prof. Stepan G. Shiyatov, IPAE URAN)

**Fig. 4:**
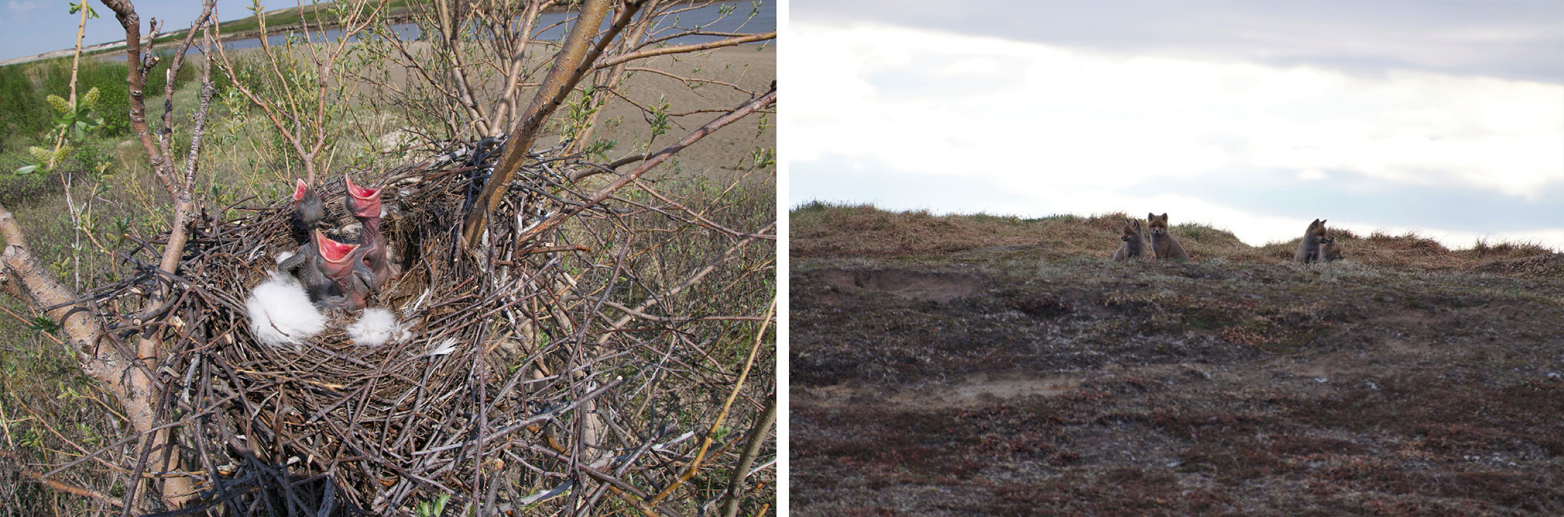
Observed effects on the environment include changes in species abundance. Here, breeding of crows and red fox at 68° N. Photos: A. Sokolov (2014)

The observed effects on the environment include changes in species composition (Kharuk et al. 2006; Magomedova et al. 2006; Shiyatov et al. 2007; Frost et al. 2018), abundance of animals and plants (Kharuk et al. 2007; Shuman et al. 2011; Epstein et al. 2018; Figs. 3, 4), species growth and distribution patterns (Sokolov et al. 2017) and annual biological cycles (Forbes et al. 2016).

Other environmental changes relate to permafrost thaw (Anisimov and Reneva 2006; Pavlov and Malkova 2010; Schaefer et al. 2011; Chadburn et al. 2017) and its impacts on epidemiological situation (Popova 2016; Bogdanov and Golovatin 2017), changes in hydrology (Streletskiy et al. 2015), biogeochemical cycles (Karelin and Zamolodchikov 2008; Naumov 2009; Golubyatnikov et al. 2013; Zubrzycki et al. 2014) and coastal sea ice retreat (AMAP 2017a).

In addition to the longer-term trends described above, climate change impacts Siberian and global weather systems, increasing the frequency and intensity of extreme weather events. Examples are local scale hurricanes, tornados, extreme dry/wet periods (e.g. Forbes et al. 2016) and increased frequency of tundra and forest fires (Bret-Harte et al. 2013). Another example is the rapid methane release from below-ground reservoirs that can cause explosions, creating craters in the physical landscape (Kizyakov et al. 2017). This is potentially dangerous if future explosions occur under oil and gas pipelines.

### The main global consequences and teleconnections of environmental changes in Siberia

Human-induced climate change is driven by a global demand for resources and, in combination with anthropogenic climate variability and various feedback mechanisms (Cohen et al. 2012), has resulted in the observed pattern of climate change.

The speed of change observed in Siberian ecosystems is fast (see Tchebakova et al. 2010; Shvidenko and Schepaschenko 2013; Bukvareva et al. 2015) resulting in little time for communities and society to adapt. Increased frequency and magnitude of extreme events, which are difficult to predict, also make it difficult to be prepared without adequate prediction abilities and warning systems. While the impacts of climate change vary across the globe, the majority of the world’s population will experience changes in climate and weather systems and thus be affected by a rapidly changing climate and associated extreme weather events. Where populations are not directly affected adversely by climate change, they are likely to be subjected to in-migration from populations suffering dramatic changes such as coastal flooding and desertification.

Processes in Siberia with global consequences include (a) changes in albedo caused by changes in snow cover, cloud cover, vegetation type and aerosols from anthropogenic emissions and natural fires, (b) changes in biogeochemical cycles such as increased emissions of greenhouse gases resulting from changes in temperature and precipitation patterns (Pokrovsky et al. 2015; Serikova et al. 2018), (c) possible changes in the highly, annually variable “Siberian High” [climatological high pressure system: see, e.g. Marshall et al. 2016], which will impact weather across much of Eurasia and (d) increases in extreme weather events outside the Arctic (e.g. Cohen et al. 2014 among many others) linked to Arctic amplification and to the greater frequency of severe winters in the densely populated Northern Hemisphere mid-latitudes.

### Major local societal impacts of environmental change and coping strategies

Determining and mitigating impacts of environmental change on local societies require preservation of indigenous knowledge (Eira et al. 2018) and increased knowledge of coping strategies (Rakhmanova 2018). An example is the instability of reindeer husbandry livelihoods under changing environmental conditions because decreases in the abundance of lichens and other forage plants such as horsetails reduce reindeer weight and force the herders to move to new areas (Lavrillier and Gabyshev 2017). Although reindeer herding can potentially benefit by modifying herding methods to sociological and climate change impacts (e.g. Tyler et al. 2007), such as land abandonment (Eilertsen 2002), there is a general need for diversification of livelihood strategies because of the instability of traditional livelihoods such as reindeer husbandry and fishing, and the risk of losing some plant species which are used traditionally for food and medical purposes. Strategies also need to be developed to cope with new pests and diseases (affecting both reindeer and people) that are likely to be introduced as a result of climate change, increased exchange of goods and human movement.

In contrast to adverse changes for local societies to cope with, there are, and will be, new opportunities, for example the introduction of new cultivated plant species. Changing climate may allow for the production of more southerly crops and hence offer an opportunity for diversification of livelihoods. However, new coping strategies should not be at the expense of traditional systems.

### Communication with local communities

The researchers acknowledged that local and indigenous peoples (especially those relying on natural resources) have an experienced and inherited knowledge of their environments and ecosystems and of local society. Although some knowledge is being lost (Eira et al. 2018) this type of knowledge is valuable for the research community when developing and designing research and monitoring projects/programmes, or when analysing and interpreting results.

New technologies are readily adopted in some local communities, but used less in others. Where new communication tools such as Internet are adopted, this offers opportunities for the improved exchange of information between scientists and community members and for informing local communities about extreme events (an example of good cooperation between scientists, local communities and decision-makers).

In a research perspective, cooperation with local communities is important for ethical reasons, e.g. making monitoring activities of scientists more transparent for the communities and making Northern Peoples active participants within studies, rather than passive study foci for the academic community and decision-makers. Research questions and study designs should be discussed and accepted together and traditional knowledge should be part of the research projects (Joint Statement of Ministers, 26 Oct 2018). It is important to directly involve, where possible, local and indigenous peoples in environmental monitoring programmes through a community-based research approach and through citizen science programmes. The information obtained needs to be disseminated further to schools, decision-makers and the general public so that local communities can learn about the required diversification of livelihood strategies and treatment of illnesses, etc.

### Communication with decision-makers

Local communities and scientists possess knowledge of the natural environment and society that can be used by decision-makers in adaptation processes, whether these are caused by climate change or globalization, and decision-makers can, through funding opportunities, shape research and monitoring programmes to address local, regional, national and global concerns.

In a research perspective, cooperation with decision-makers is important for making decision-makers aware of changes in climate, ecosystems, environment and society and the impacts of these changes. It is also important to ensure that research and monitoring programmes are relevant for societal challenges perceived by local peoples and decision-makers. Cooperation with decision-makers also provides a platform to lobby for funding of prioritized research and monitoring topics.

### Recommendations from scientists to improve the efficiency of working together

The dialogue reported here among local and indigenous peoples, decision-makers and scientists was partly facilitated by INTERACT (www.eu-interact.org), a network of 86 research stations in 18 countries including all the Arctic nations. The network was often considered (below) to be a vehicle for implementing many of the recommendations. The recommendations from the scientists were as follows:

- There is an obvious need for fundamental science to advise best practice under future environmental conditions.
- *Coherent* messages from scientists to decision-makers are essential and these could be produced by using standardized circumpolar data sets.
- Cooperation between research stations and local people should be improved. INTERACT will contribute by providing a printed book for station managers on appropriate and successful practices for engaging with local people (see 3 examples briefly summarized in Table 1 and in Electronic Supplementary Material S1).

**Table 1:** Case studies highlighting three key issues where scientists, local and indigenous peoples and local decision-makers need to work together to ensure sustainable Northern futures

Case study	Area	Key stakeholders	Key issue	Key processes	Recommendations
1. Living resources of the Southern Taiga	River Ob flood plain, western Siberia	- Regional Administration - Hunters and Fishermen - Universities - Private farmers - State collective farmers	Interaction between fishing and hunting livelihoods of local people on one hand, and conservation regulations on the other	- Beaver population growth - Fishing quotas	Develop a new legislation based on informed recommendations from the research community and land users, e.g. hunters fishermen and conservationists Ensure a fair and transparent decision-making process through dialogue among stake holders
2. Pressure on reindeer pastures in the Russian tundra	Yamalo-Nenets Autonomous District, Siberia	-Reindeer herding enterprises -Individual reindeer herders and their families -Local Government - Oil and gas industry	Interaction between indigenous peoples traditional livelihoods and general development (e.g. infrastructure) in the area	- Increased numbers of reindeer - Reduced and fragmented pasture areas	Calculate the current reindeer capacity of the pastures from a geobotanical survey Encourage local and federal authorities to provide essential regulation and legislation with the help of Nenets (and other) herders to ensure sustainable reindeer husbandry
3. Reindeer herding and tourism in Scandinavia	Northern Scandinavia (sub-Arctic)	- Reindeer herders - Herding authorities - Research institutions (Stations and Universities including Tromsø, Umeå) - Forest industry - Mining industry - Hunting and fishing associations - Conservation authorities - Tourism businesses	Interaction between traditional land use (reindeer husbandry) and increasing tourism business	- Reduced and altered snow season and poor winter conditions in the European Alps - Increased tourism activities in the North - Decreased opportunities for winter pasture for reindeer and increased disturbance to herds	Ensure that tourist operators, local and indigenous peoples have good dialogues (use existing good example between reindeer herders and the operators of the Finnmarkløpet as a model for other tourist activities) to advise local decision-makers on actions needed to ensure sustainable tourism and reindeer husbandry in the area
- Protocols should be published so that, for example new research stations, projects and individual local communities can interpret their local data in a pan-Arctic context.
- The INTERACT Station Managers’ Forum, where station managers meet, can serve as a place for training and sharing best practices.
- Support and funding is essential for further development of environmental monitoring networks throughout the vast and environmentally diverse Russian Arctic to inform local decision-making. It would be highly beneficial to launch a new network of research and monitoring stations in the Yamal-Nenets Autonomous region of Russia that has the advantage of being linked to the wider Siberian Environmental Change Network, the pan-Arctic INTERACT and numerous other relevant single-discipline networks.
- SecNet stations should also be included within INTERACT to unify the protocols that are used in monitoring and research, and the networks should be widely advertised for Russian institutions to join.
- The combined networks can serve as an early warning system for potentially hazardous events building on the one-stop-shop initiative developing within INTERACT.
- Transporting samples taken from the field and sent across national borders for analysis should be made easier by reducing and/or removing the barriers of cross-border import/export.
- Global ethical concepts should be adopted by any new stations from the Yamal-Nenets Autonomous Region, SecNet and INTERACT.
- Achievements of Russian science, and successful interfaces between this science and local and indigenous peoples and decision-makers, are mostly invisible for the international society. The process of integrating these achievements into the global community is not fast, and a greater coordination of information flow should be introduced. This could be done via, for example a Science Forum, where the research opportunities in the Russian Arctic and dialogues among researchers, local peoples and decision-makers could be promoted.

## Issues perceived by local peoples

### Background to the contribution of information from local communities

Representatives of different Siberian indigenous peoples living on the territory of the Yamal-Nenets and Khanty-Mansiysk Autonomous districts, Yakutia, Chukotka, Murmansk and the Arkhangelsk Region were willing to discuss urgent problems. In addition, indigenous peoples from Norway, Finland and Sweden were invited to provide their perspectives and discuss common issues. However, the methodology and format of the science-based workshop sometimes caused them to be perplexed, because they were accustomed to a different way of generating ideas and decision-making. Differences in the tactics of the discussion were also noticeable between English-speaking reindeer herders of Scandinavia and reindeer herders of Siberia, who built a dialogue through an interpreter. Also, the group consisted of local people who have received higher education and are engaged in counselling and assisting representatives of indigenous peoples. This participant structure shows that the model of discussion was initially set in a paradigm that is close to Eurocentric, which sets a dialogue with people from the “nomadic world” within the realities of European standards of consumption, health care, transport systems, communications and life support in general.

### Issues relating to health and well-being of local populations

The participants were immersed in environmental and management discourses to a varying degree, and their statements represented different levels of understanding of the proposed problems. The discussion was, therefore, a result of a combination of scientific and “everyday” logic, manifested from the “inside” of traditional culture. The perceptions of issues related to health and well-being varied between the Scandinavian and Russian reindeer herders in some cases as their national infrastructure and economy lead to different living conditions. However, there were shared concerns on some issues.

Russian reindeer herders, practising reindeer husbandry in the Yamal-Nenets Autonomous District, recognized several key issues related to their well-being and health including the absence of frequent ambulance flights, the importance of maintaining traditional food sources, the depletion of pastures for reindeer (as meat is the basis of their diet), and for local people from settlements where fish is the basis of the diet, the problem of declining fish stocks in rivers and lakes. The final key issue identified was a declining immune system as this is weakened when the centuries-old “diet” and eating habits change under the influence of changing anthropogenic and natural factors.

Scandinavian reindeer herders, practising reindeer husbandry in northern Norway, Sweden and Finland, commented that the main cause of the imbalance in the lives of Northern indigenous peoples compared to the well-being of people from cities is that indigenous knowledge is not included in the scope of informational resources for the process of decision-making by governing structures and is, therefore, not part of the public debate *(“Knowledge of indigenous people is not included in the structure of discussion by the decision makers”).* This observation emphasizes the need for indigenous peoples to have a greater dialogue with decision-makers and a greater role in decision-making processes that affect their well-being and also the need to protect indigenous knowledge as a “living” resource (Eira et al. 2018).

Both the Scandinavian (Saami) and Russian (Nenets, Khanty and Yakuts) participants shared the views that mobility with migrating reindeer herds and nomadic lifestyle are key factors for well-being and both physical and mental health. It was perceived that the abrupt transition to a settled way of life in villages and towns is leading to psychological instability (L. Rakhmanova, pers. obs. based on interviews), reduced immunity and more frequent cases of diseases of various kinds, for example cardiovascular diseases and diabetes (Rautio et al. 2014). The transformation from nomadic to a sedentary way of life may also lead to psychosomatic disorders. A sedentary lifestyle can lead to obesity, particularly in men, but also in women which affects the reproductive health of the population. Also, the change of the rhythm of life and the resulting free time, atypical for the nomadic way of life, leads to alcohol abuse among some Northern Peoples (Lobanov et al. 2017; Martinchik et al. 2017). Equally important to the well-being of the local population is the safety and quality of food and water and the control of its handling, transport and storage, i.e. food security.

Perhaps the most significant integrator of the issues documented above is the life expectancy of the indigenous peoples of the Arctic taiga and tundra zones. Life expectancy and mortality rates are the indicators that are most easily captured and recorded by community members themselves, and therefore, they begin to “sound the alarm” even before the demographic statistics recognize any crisis situation (Rautio et al. 2014). According to the indigenous peoples of the North, especially in the territory of the Russian Federation, life expectancy is decreasing because of the declining quality of their lives, provoked by an attempt to apply to the Arctic realms the criteria developed for social policy in milder climatic conditions. According to interviews with local residents (Kargasok district of the Tomsk Region, Nizhnevartovsk and Surgut Districts of the Khanty-Mansiysk Autonomous District, Nadym, and Purovsky Districts of the Yamal-Nenets Autonomous District), there is an increased anxiety about the life expectancy of the populations. However, local perceptions of living conditions, health and longevity differ from the existing statistics that show an increase. This contradiction can be explained by two factors. First, health and life expectancy are linked by local people directly to environmental conditions. Despite the increase in the quality of medical care in these regions, trends from the position of local residents show deterioration in health and a decrease in the number of long-lived people. Secondly, during the interviews, we found out that the term “life expectancy” is interpreted by ordinary people in a slightly different way compared to the interpretation used by municipal administrations and scientists: they believe that long-lived people are the ones who can perform various functions: economic, social and family, until their last days. Therefore, although life expectancy has actually increased in the regions, the period of active life of citizens, especially men, has decreased. Added to the concerns over life expectancy is a change in demography because in the taiga, populations are decreasing due to abandonment of agricultural land and villages and a movement to towns and cities.

### Perceptions of issues related to environmental and climate change

Ongoing climate change in Siberia and northern Scandinavia results in abnormally hot summer seasons and floods that increasingly affect coastal communities. These changes, in turn, have led to a successful invasion of rare species of insects that have been absent before. The growing populations of horseflies and mosquitoes have a significant impact on tundra and taiga reindeer husbandry. In addition, newly arrived birds and insects may transmit new viruses and diseases from southern regions. These may be particularly dangerous to inhabitants of the Northern regions who do not have appropriate immunity (Popova 2016; Bonebrake et al. 2018; Waits et al. 2018).

Ongoing climate change also affects Arctic biodiversity through species increase, decrease, immigration or emigration. Resulting dramatic changes in the migration patterns of wild animals, birds and fish change the fishing, hunting and conservation practices of local populations (see Case Study 1 in Table 1 and Electronic Supplementary Material S1). Biodiversity is also affected by the increasing human presence in the Arctic that results in niche loss, habitat loss and habitat fragmentation for many species (e.g. Taran 2018).

The observed changes in snow conditions in the Arctic and, concurrently, in the southern regions, have many impacts in the North. Decreasing snow cover in the South leads to an increasing demand for Arctic tours. This, in turn, is triggering a *“tourist invasion”*. Snowmobile and road trips in sparsely populated areas of the Scandinavian Peninsula cross the paths of herders’ migrations and invade the historically established areas of intensive traditional land use (see Case Study 3 in Table 1 and Electronic Supplementary Material S1). In addition, there is a threat of fragmentation of nomadic cultural space caused by the sale and long-term lease of the land on which reindeer pastures are located. These changes are supported by new land legislation regarding land rights in disputed territories, for example the number of concessions given to the extractive industry sector in disputed territories has increased, especially in Sweden and Finland.

One global result of climate change was articulated by a Saami reindeer herder who said that *“a positive effect of climate change is that the world community has finally focused on the needs of indigenous people”* and, in fact, on the Arctic in general.

### Expectations of local peoples on scientists and interactions between them

**Fig. 5:**
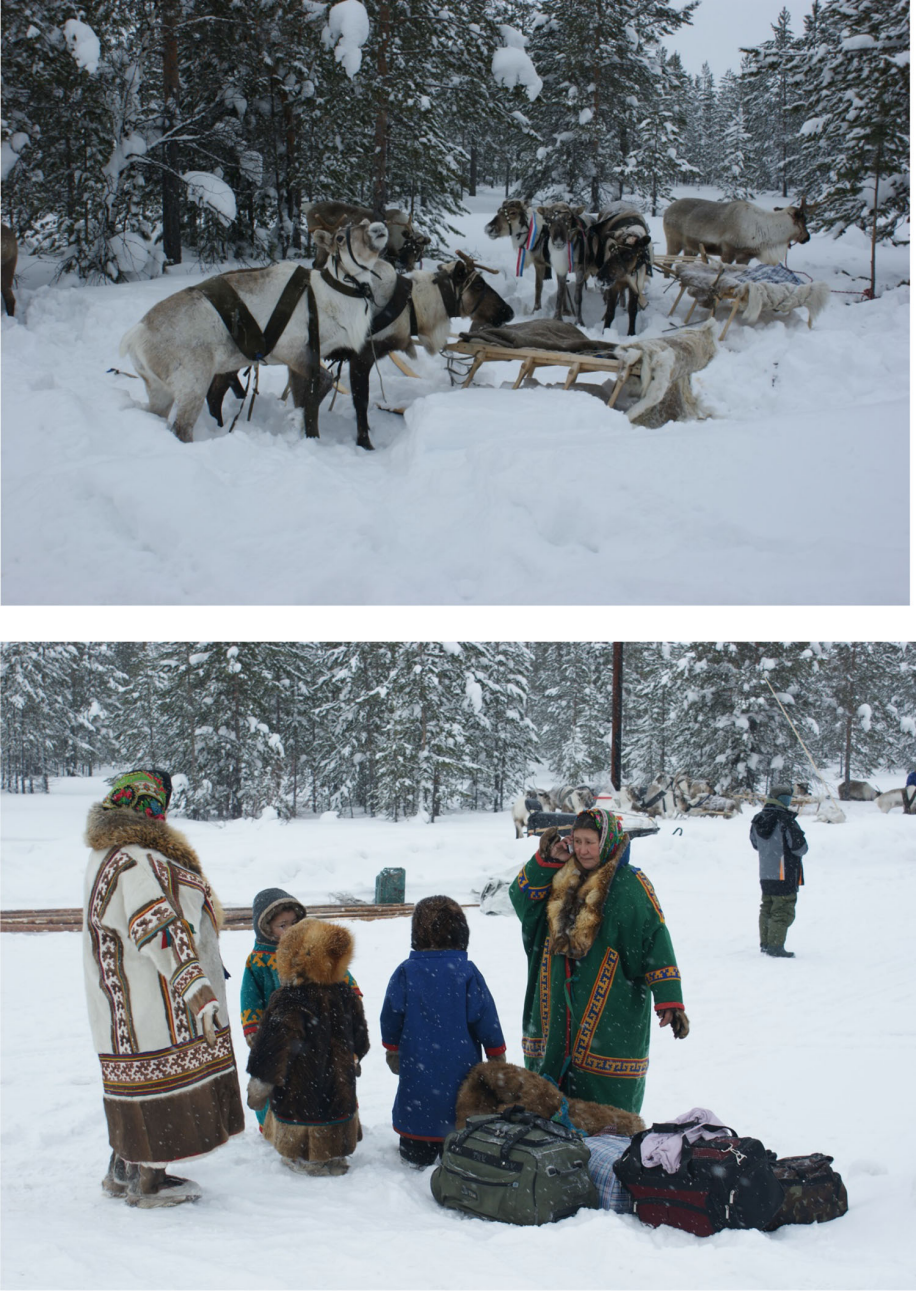
People of the North are affected by climate change and globalization. For example, Khanty People keep many of their traditions such as use of reindeer but are part of modern society using mobile phones, snow scooters, etc. (Photos: S. Kirpotin)

History has shown that communities are able to adapt to past and present changes in local environments (Fig. 5) and ecosystems, as well as to the influx of external societal development processes and technology, although the changes are not always desirable for all the community members [e.g. western diet (Harvald and Hansen 2000)]. In some cases, new technology opportunities are quickly adopted by many (e.g. mobile phones, GPS, snow scooters, radio frequency identification for reindeer tagging [NIBIO 2016 Meld St. 2016–2017], but in other cases local and traditional knowledge systems are no longer adequate or relevant for facilitating adaptation to current changes (both environmental and globalization). In this latter case, input from scientists is essential. However, field studies by scientists from different disciplines can be considered by local residents in two basic ways: as an invasion into their culture and everyday life and/or as an activity that brings them new information, opens up new perspectives and helps them to adapt to the changing environmental and socioeconomic conditions of life in the Arctic.

### Recommendations from local and indigenous peoples to improve the efficiency of working together

- A “translation” of scientific language is required to make scientific knowledge clear and understandable to the indigenous population of the Arctic (“*let’s make science language clear and understandable*”). It is possible that one of the steps to solve this problem of “translation” could be the appearance of a new generation of scientists from the communities of indigenous peoples. The most effective and most demanded types of specialization in education for young reindeer herders and their families are medicine and veterinary science.
- Researchers often depend on accessing lands occupied by local peoples and they often receive help and information. However, far too often, researchers leave behind disturbance and research artefacts but not knowledge. Feedback from scientists from their work should be a pre-requisite for their access to the field. Information to local communities from long-term field research would be particularly important, and scientists should provide the most relevant data to the inhabitants and in an understandable way. For example, information on endangered and rare animal species and other natural resources that should be protected to maintain a balance in the ecosystem for the benefit of future generations could be very useful. Other helpful data that were identified were weather reports, long-term weather/climate projections, forecasts of the dynamics of water levels in rivers and access to economic forecasts that are important to maintain livelihood stability.
- It is also recommended that, in parallel, researchers should request advice from experts among the indigenous population *(“feedback from the locals to scientists”)*. This means that local people should no longer be the “object” of research, but rather a participant in the research process. The principles of research ethics suggest that researchers should be “prepared” for interacting with local people before going into the field in order to avoid their own culture shock, and so as not to cause trauma due to this intervention to the local community. A deeper form of partnership with the local residents should involve the joint formulation of research questions and hypotheses to make research more relevant to local peoples. Such practices are well developed in the Canadian and USA Arctic (Hitomi and Loring 2018) and are presented in “Principles for the Conduct of Research in the Arctic”, https://www.nsf.gov/geo/opp/arctic/conduct.jsp).
- Local peoples did not focus on mechanisms of improving dialogue with decision-makers but rather focused on various forms of action and support they required from decision-makers at regional and federal levels of government. There was an indication of some misstrust between the groups because local peoples perceived that some aspects of current dialogue between local populations and administrations were failing. An example offered was conscious or involuntary misinformation to the population about ongoing natural disasters, emergencies, economic crises and changes, and changes in social policy.
- Decision-makers should take an intermediary position of communication between scientists and local people. Decision-makers can also develop an organizational infrastructure that enables the process of mutual tripartite consultation of the stakeholders (local population, scientists and managers) and a greater role for local peoples in decision-making processes.
- Decision-makers in Siberia should strive to over-turn the inequality in the distribution of benefits, subsidies and other resources between “sovhozes” (state farms owning reindeer herds left from the Soviet times) and individual reindeer herders who are often deprived of their rights and opportunities which are instead granted to “brigades” (groups of herders taking care of state-owned reindeer for a fixed salary).
- Government and municipal authorities should also strive to over-turn the perceived inequality in the support for Nenets who dominate the ethnic groups of Yamal (which is a multi-ethnic region). Other ethnic groups such as Khanty and Selkups apparently receive less support.
- Environmental consciousness in the Arctic zone requires legislative regulation of the quotas and methods for hunting, reindeer husbandry and fishing. An important issue is to find a balanced way of developing reindeer husbandry while maintaining its uniqueness and balancing legislature with local flexibility.
- Decision-makers are encouraged to shift the focus on energy sources from existing fossil fuels to alternative energy sources. From the reindeer herders’ perspective, this would slow down the destruction of the habitat for reindeer and other Arctic animals and plants. From the researchers’ perspective, it would reduce the level of global greenhouse gases.
- To ensure that Northern indigenous peoples’ way of life can be protected and preserved, current regulations need to be revised as regulation for some species of animals will no longer be appropriate in a changing climate.

## Issues perceived by decision-makers

### Major challenges for sustainable development

A working definition of sustainable development *“Development which respects local environment and local people with long term viability prioritized over short*-*term gain”* was used to identify several issues. A current aim of the government of the Russian Federation is to increase the population of Northern regions (Eremenko 2014; Fauzer 2014; Gagiev 2016; Kiseleva and Gokova 2016) but firstly, there should be an assessment of how this policy would affect the environment. Furthermore, cost implications of settlement should be considered. When starting new industrial projects, especially in the high Arctic, an important decision should be made between encouraging settlement by shift workers or settlers with families as sustaining the life of each individual is extremely expensive (Efremov 2014). This expense originates from providing a degree of “comfort” appropriate to inhabitants in the world of modern city-dwellers. However, there is a dilemma: what degree of comfort should be provided for indigenous nomads? On one hand, when planning the modernization of local peoples’ lives, they should not be forced to change their lifestyle whereas they should have access to the advances of modern medicine and education. Providing “modern facilities” has increased infant survival and life expectancy in the tundra-living human population. An increasing population leads to new issues that need to be solved such as the danger of reindeer pasture overgrazing in the Yamal-Nenets Autonomous District (See Case Study 2, Table 1 and Electronic Supplementary Material S1).

### Opportunities and challenges for communicating with local and indigenous peoples

Local and indigenous peoples perceive current issues from a very long-term perspective as their ancestors have been living in the tundra for centuries. Now they see how companies come to drill for oil and gas on their native lands. Even if they are not harmed by this intervention (Forbes 2013), local people understand that companies are making large profits and local people want to have their fair share. As a rule, companies are not against a dialogue, but the local and indigenous peoples should know from the beginning exactly what they want.

In such dialogues, there is an inequality of negotiating positions, as both sides see the other as a potential opponent and do not understand the way of thinking of their “opponent”. Furthermore, each side has a very different level of empowerment from which they can influence the situation, the local peoples having a much weaker power base than the decision-makers. This fundamental division is amplified by divisions within the local communities. Representatives are chosen from the local communities and sent to negotiations where they become known by Government and are elevated to a privileged sector of their former local community. This leads to separation and perhaps alienation from the rest of their original community. After this stage has been reached, it is questionable if the Northern People’s representatives faithfully represent their local communities. This situation has an analogue among the decision-makers when representatives of local government interact at the Federal level.

The decision-makers identified that “Talent loss” hinders dialogue with local communities. Talented young people from local backgrounds that leave to get higher education in large cities often fail to return to their homeland so there is a lack of educated people within local communities to interact with decision-makers.

A complex question decision-makers have to be ready to answer in the dialogue with local and indigenous peoples is what happens with the Arctic territories after oil and gas reserves have been exhausted. Which types of development should be used then to sustain the current quality of life resulting from the high profits from the previous exploitation of carbon deposits?

### Opportunities and challenges for communicating with scientists

The Government has the difficult choice of assigning funding priorities to applied science that could solve important, urgent problems or fundamental research, hoping to have exciting results in the future. Specialists coming from the ‘big land’ outside are not aware of the local specific conditions, so they are harder to communicate with. According to federal law, environmental impact assessments should be carried out for all construction work or mining activity. However, the quality of such assessments is not always satisfactory, and the companies rarely, if ever, share the results with the scientific community who could comment on the adequacy of the assessment and the weakness of field work or analysis.

There is a lack of locally trained scientists in the Russian Arctic. For example, there is no University in the Yamal-Nenets Autonomous District. Local science exists in some of the Arctic regions of Siberia but it is hard for the institutions concerned to develop in the same way as large federal scientific institutions. Also, international research programmes tend not to collaborate with small, local institutions, failing to share results and not involving local specialists in the field work.

There is a need to develop a coherent message from scientists as to what scientific monitoring priorities are. A good example of when the global scientific community set out to measure one clearly defined parameter that leads to a clear result, and is therefore well-funded, is the CALM (Circumpolar Active-Layer Monitoring) programme (Brown et al. 2000).

### How should decision-makers respond to current and likely future hazardous events?

Fundamental science is needed to advise best practice under future environmental conditions as hazardous events are becoming more frequent. Examples are strong blizzards late in the season, and extremely hot summers that facilitate the spread of spores of Anthrax (see Case Study 2 in Table 1 and Electronic Supplementary Material S1). We still cannot predict these events in advance. More knowledge-based decision-making is needed to identify and respond to such events. Hence, support and funding for monitoring networks are needed in the Russian Arctic to inform decision-makers on likely future hazardous events, preferably in cooperation with the INTERACT “rapid response to potential environmental hazards” activity.

Building regulations need to be updated to account for the heterogeneity in local conditions and future change, as current ones are based on 1980s knowledge. Permafrost thawing is becoming a real problem for pipelines and buildings (e.g. “new” (< 10 years old) tall buildings are already facing challenges).

### Recommendations from decision-makers to improve the efficiency of working together

**Fig. 6:**
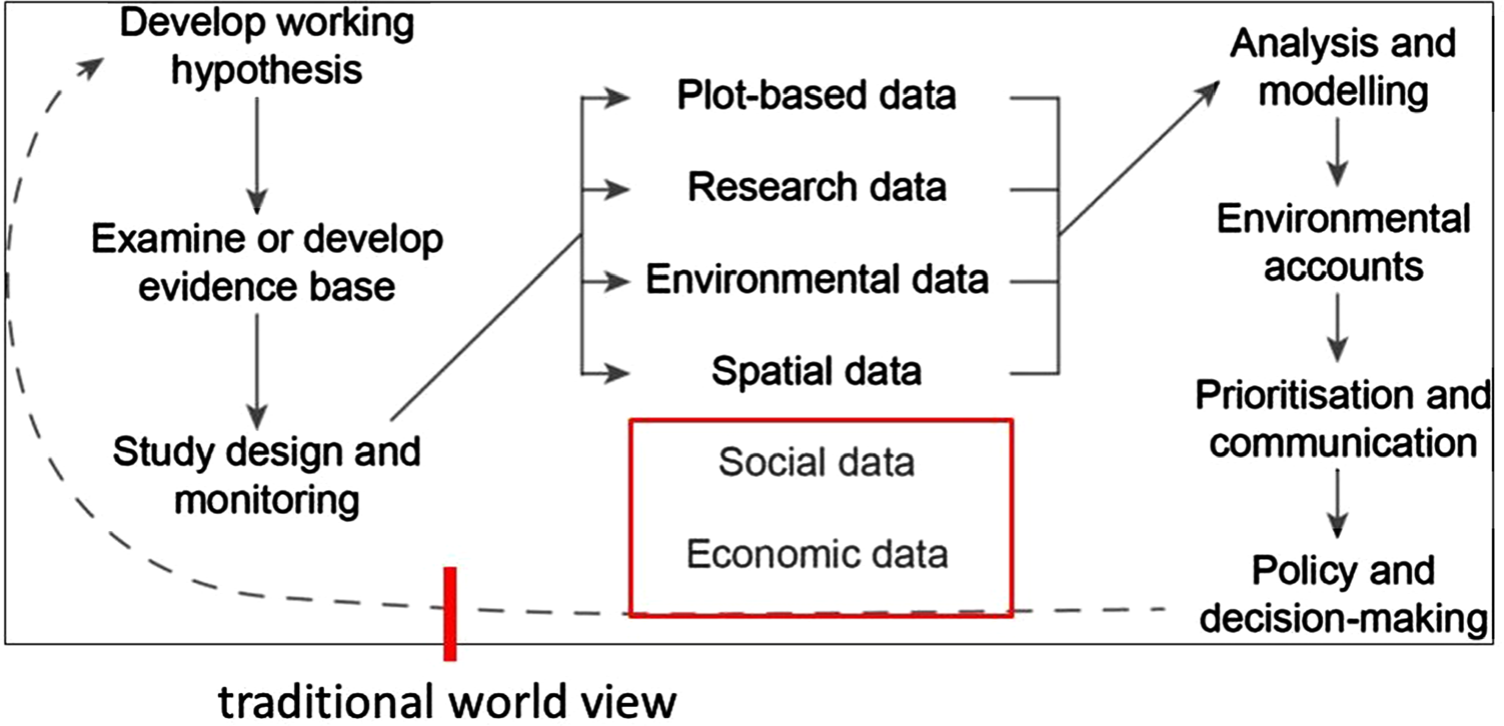
The critical barrier in a conceptual model highlighting key linkages between monitoring and environmental reporting (Lindenmayer et al. 2015)

- To achieve improved communication, all three groups should use a language that is understandable for everyone, e.g. by avoiding jargon.
- Training opportunities should be provided for scientists, local and indigenous peoples and policy-makers to learn to speak each other’s languages. Scientists should learn to understand legal frameworks and how the government systems work in order to write policy briefs; decision-makers should learn the basic principles of research and the way the local and indigenous peoples’ lives are organized; local and indigenous peoples should learn in which way science could help them and also their legal rights.
- Each group should be as specific as possible regarding what they need from the other groups.
- Funding should be found to support the training of local and indigenous peoples to work as rangers on their land and this would also be beneficial for scientists, e.g. by looking after scientific equipment for a small salary.
- The trust between local and indigenous peoples and other groups, particularly policy-makers, researchers and the media, should be increased keeping in mind that trust can only be established by long-term contact.
- At a wider geographic scale, one of the obstacles hindering international collaboration is the difficulty for foreigners to access the Arctic part of Russia as it is considered “a border zone”. Requests for a visit should be sent two months in advance and it is not always possible to plan ahead. It would help if decision-makers could make this procedure easier for visiting scientists.
- An established scheme, e.g. by Lindenmayer et al. (2015) should be used to improve dialogue among the various actors (Fig. 6).

## Three case studies of collaboration between researchers, local and indigenous peoples and decision-makers

Three case studies of collaboration between the groups have been carried out and are summarized in Table 1. Full details are found in the Electronic Supplementary Material S1.

## Overall conclusions

Ongoing climate change and societal development in Siberia and other northern areas affect local communities and livelihoods as well as general developments in society. The ongoing and projected rapid changes in natural systems (climate and ecosystems), extreme events, new pests and deceases, and new opportunities (sea transport routes, crops, tourism, etc.) all put pressure on society to adapt—from individuals, households and communities to regional, national and global institutions.

A communication platform for information exchange and cooperation is required for the dialogue between local and indigenous peoples, decision-makers and scientists. Such a platform can be used to develop potential environmental hazard detection systems, discuss research and monitoring priorities and discuss adaptation needs that benefit all groups.

**Fig. 7:**
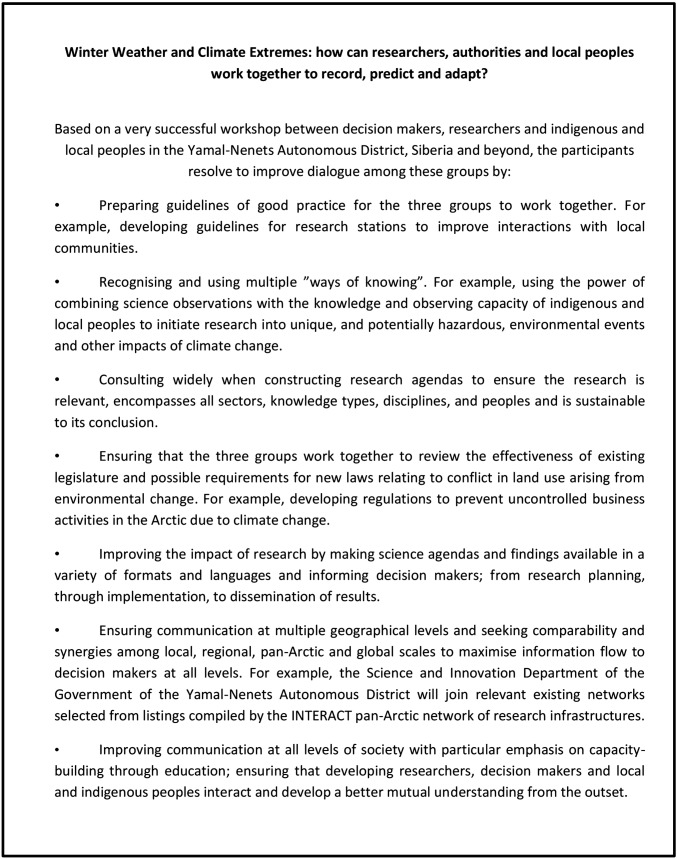
Siberian Environmental Change Network 2017 Workshop Resolution

Bringing indigenous peoples, decision-makers and researchers together has started this process at a small-scale and has led to a new state-of-the-art understanding based on different perceptions of similar phenomena and a dialogue has been established among the three groups resulting in an agreed resolution (Fig. 7). It is important to highlight that the representatives of the different groups have completely different perceptions of the events happening in everyday life so hardly ever understand each other in official dialogue. The format of the workshop described here forced the different groups to listen to the other side, giving participants insights into the other groups’ ways of thinking and thus helped to overcome perceived obstacles to effective communication between the groups. Furthermore, the study is state-of-the-art in making perceptions from discussions among Russian decision-makers, indigenous peoples and researchers globally accessible.

Since the meeting, all three groups have worked together successfully to achieve some of the aims and recommendations presented here: indigenous peoples are working with scientists and decision-makers to establish new research stations and environmental monitoring sites, and a citizen science programme is developing centred on Nadym that involves indigenous peoples, scientists, health workers and oil and gas employees. Hopefully, these small steps will multiply quickly.

## Important note

During the preparation of this manuscript, a few participants withdrew because they believed (a) “Stimulating (and sponsoring) local people to live in XIX century and use their traditional way of life in these days of technologies is simply not appropriate”. (b) “In case of on-going climate warming, there will be only BENEFITS for Russian (mostly Siberian) economy”. (c) “Most of on-going ecosystem changes are NATURAL as they did occur in the past”. (d) “THERE WILL BE NO REAL increases in concentration and fluxes of carbon and related elements in case of on-going climate warming and permafrost boundary shift northward and the increase of the thickness of the ‘active’ unfrozen layer due to permafrost thaw”. (e) Some of the statements regarding indigenous peoples and reindeer grazing were seen as controversial. These statements emphasize the need for both greater interactions between researchers and local peoples and researchers with different interpretations of current climate change and its impacts. The meeting that led to this study and opposing views will hopefully stimulate a continuation of a critically important dialogue both within and among the three groups.

## Electronic supplementary material

Below is the link to the electronic supplementary material.

Electronic supplementary material 1 (PDF 751 kb)
