# Supplementary material for: Improving dialogue among researchers, local and indigenous peoples and decision-makers to address issues of climate change in the North
Source: Ambio. 2019 Nov 12;49(6):1161–78. doi: 10.1007/s13280-019-01277-9 (PMC7128002; doi:10.1007/s13280-019-01277-9)
Supplement: Supplementary file 1 — Electronic supplementary material 1 (PDF 751 kb) [file 13280_2019_1277_MOESM1_ESM.pdf]

Ambio

Electronic Supplementary Material

This supplementary material has not been peer reviewed

Title: **Improving dialogue among researchers, local and indigenous peoples and decision makers to address issues of climate change in the North**

THREE CASE STUDIES OF COLLABORATION BETWEEN RESEARCHERS, LOCAL AND INDIGENOUS PEOPLES AND DECISION MAKERS

Case study 1. Living resources of the Southern Taiga

Area: River Ob flood plain, Western Siberia (Fig. S1)

Coordinates: N57°14'45,38" E84°11'06,17",

Environment: The climate is temperate continental. Average annual temperature: - 0.8 ^{o} C, average January temperature: -17.1 ° C, average July temperature: +18,7 ° C, average annual rainfall: 482 mm.

Research Station: The Kajbasovo Research Station is operated by Tomsk State University. It is located in the floodplain of the River Ob, which is one of the largest in the World. The floodplain (Fig. S1) is the richest landscape in Siberia in terms of productivity and biological diversity. The vegetation cover of the River Ob floodplain is represented by different types of large-scale herb meadows, by swampy-meadow phytocenoses and by floodplain forests. The terrestrial and aquatic fauna is diverse with some species important for hunters, some important as Red Book species and some, such as sturgeon (*Acipenser baerii)* and white salmon (*Acipenser transmontanus)*, economically valuable.

Population: In 5 BC. – 5AD, the Kulayskaya Culture developed in the forest zone, from which developed the nations - Selkup, Khanty and Siberian Tatars which inhabit this territory now. The face of the Tomsk Region started to change considerably with the migration of Russians who

now make up 88.1% of the population of 1,047,394 (Russian Census for 2010). The population density is 3.4 persons per km^2(http://tmsk.gks.ru/wps/wcm/connect/rosstat_ts/tmsk/ru/statistics/). From a clearance and settlement of areas of forest by Russians in the Soviet times, there has been a post-Soviet tendency for abandonment of agricultural lands and return to cities, particularly since the 1980's.

The leading sector of the economy is agriculture but the main wealth of the area in which the station is located is forests with significant reserves of wild plants, mushrooms, nuts and berries which the local people actively collect and sell.

Key stakeholders:

- Department of Natural Resources and Environmental Protection of the Regional Administration, Tomsk Oblast
- Tomsk Regional Public Organization Tomsk Regional Society of Hunters and Fishermen
- Tomsk State University
- Institute of Soil Science and Agrochemistry of the Siberian Branch of the Russian Academy of Science, Novosibirsk
- Forest Institute of the Siberian Branch of the Russian Academy of Science, Krasnoyarsk, Novosibirsk Branch
- Private farmers
- State collective farmers

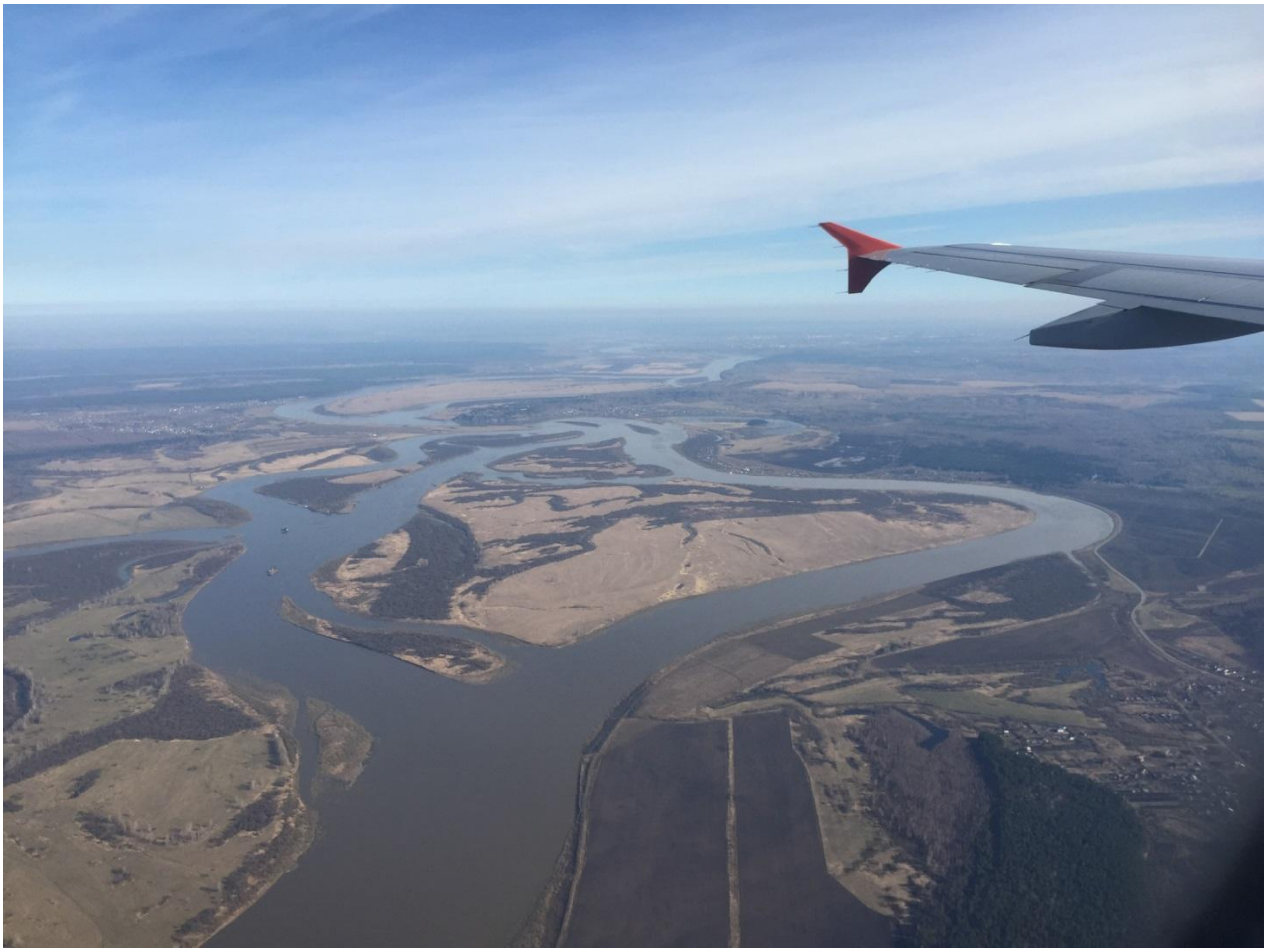

Fig. S1. Part of the floodplain of the River Ob in the southern Taiga showing rivers, wetlands, forest, cleared forest for agriculture and a settlement. Photo: T.V. Callaghan

Background

The region in this case study is rich in natural resources and provides many ecosystem services. The area is rich in gas and peat deposits, has extensive forests, grasslands for hay production, and wetlands, and is rich in wildlife such as fish and game. This case study will be restricted to the interaction between fishing and hunting livelihoods of local people on one hand, and conservation regulations on the other.

Aim

To highlight shortfalls in the development of past regulations on hunting and fishing and to recommend a more responsive process more appropriate to a rapidly changing environment and way of life.

Existing process

Beavers have spread in the Tomsk Region (as in North America and UK, (Halleyl and Roself 2003; Puttock et al. 2017) from very few 20 years ago to a large population that now adversely affects the ecology of water bodies. These adverse effects include significant reductions in the diversity and volume of fish resources throughout the Tomsk Region (Popkov et al. 2017), tree felling which is undesirable to landowners, and dam building that could result in floods of forest, farmlands and roads. These problems have developed mainly because beavers have been managed as a protected species. Similar consequences of administrative decisions that had a beneficial result 50 and 60 years ago, but now are resulting in adverse impacts, can be observed in the European part of Russia.

Local authorities need to amend local legal acts regulating the numbers of animals, in particular, the number of beavers (*Castor fiber)*. This is because the original rules have been made irrelevant. However, local authorities sell hunting licenses for some animals such as wolves (*Canis lupus*) and bears (*Ursus arctos)* and the number of licenses sold is based on animal population numbers. Over the past decades (30-50 years or more), the extraction of natural resources and everyday life in remote villages have changed significantly. Economic trends (local, regional and global) and adaptation to this changing reality have driven decreases in the populations of local people and a lack of demand for pelts. Consequently, today, neither skin

nor meat of beavers attract local hunters, resulting in an increasing population of a currently (inappropriately) protected species and greater environmental damage. Such societal changes can affect the physiological well-being of local populations as well as their economy. Balancing conservation needs, local economy and environmental protection is an activity that requires researchers, local peoples and decision makers to work together.

The activities of hunters and fishermen are not just related to market conditions. Their activities are also influenced by the type of control and the severity of sanctions for violations which play a significant role in environmental management. It is control that influences the way of life and the type of economy of local residents of remote areas more than the market. Although the scientific study of populations of animals and fish, and the state of water and soil are the basis on which justifiable decisions should be taken when developing legislation for the protection of the environment, decisions are made by the officials often without connection to the data obtained from the academic community (Rakhmanova 2018). The scientific basis for sustainable use of resources is not used to give everyone equal access to a certain natural resource, but to prohibit extraction by certain groups and to grant this right to other groups and communities. Thus, the indigenous inhabitants of these territories may be branded from a legal perspective with the status of poachers for years, while their actions from a humanitarian perspective could be assessed as a search for ways to survive.

Fishing in the Tomsk Region is practiced at an industrial scale by nets in fish farms operated by private consortia or middle-size companies cooperating with local authorities, and for private needs (recreational fishing) by rods in the Rivers Ob and Tom by local residents (nets are prohibited to conserve biodiversity and stocks). The Tomsk Region is the only region in Western Siberia where recreational and industrial sterlet (*Acipenser ruthenus*) fishing is allowed. The

local government is establishing new fish farms to increase fish populations. This process unfortunately is not going smoothly. A few years ago, the Tomsk Region and Khanty-Mansijsk Autonomous District made an agreement about the provision of juvenile valuable fish to the Tomsk Region. From the small local fish-business prospective this decision could not be economically effective in a long-term prospect and it caused some tension between different types of regional stakeholders.

Industrial fishing processes are governed by Federal law, and the rules do not change spontaneously, but are planned, taking into account various perspectives. For example, industrial users in the Tomsk Region received quotas in 2008 for sterlet, peled (*Coregonus peled)*, muksun (*Coregonus muksun/ Lactarius lactarius*) and nelma (*Stenodus leucichthys).* But in 2019 all contracts end. Is that the end of fishing? No, Rosrybolovstvo (Federal Agency for Fishery in Russia) renegotiated contracts with everyone who wished for another 15 years. Until 2034, users have quotas that may change every year depending on the scientific advice of ichthyologists on the catch of one species or another.

For the present recreational fishing purposes in the Region, 112 fishing grounds of 113 thousand water bodies operate where fishing is permitted by nets with vouchers from the organizer of recreational fishing, mainly the societies of hunters and fishermen. In the Tomsk Region, formerly there were rules for recreational fishing, according to which every inhabitant of the Region was allowed to fish with nets (four nets, 25 meters of drain), but there were daily allowances per person, and abuses were rare, according to the local authorities.

During the last 5 years the regulations for recreational fishery were changed several times and not in a favor of local residents or local authorities. The last draft (2018) of the Federal Law "On Recreational Fishing", excludes use of fishing grounds for organizing recreational fishing and

restricts the types of fishing equipment. Also, it plans to charge for the permission. For the Tomsk Region, where only 25 water bodies could be open for recreational fishing according to the new rules, there could be a dramatic increase in poaching catch. As for the region of this case study, there is not one official recreational fishing water body!

Recommendations

There is an explicit need for legislation to be based on informed recommendations from the research community. As an example, a hunter and fisherman from the Tomsk Region shared his observations of fish stocks and of beaver population in the long-term perspective with scientists working at the Tomsk State University research station "Kajbasovo". These observations became the basis for a large research project that will result in the development of practical recommendations for the regional administration. These recommendations may prove the need for changes in the environmental protection rules for beavers.

Different measures are required to limit the number of specially introduced fish species (e.g. bream, *Abramis brama*), like trapping reproducing adults in the spring period in places of mass spawning as an additional measure to the cancellation of fishery limits for this species (Popkov et al. 2008; Popkov 2017).

There is also an explicit need for a dialogue among stake holders to ensure a fair and transparent decision making process in the allocation of hunting and fishing quotas to all relevant stake holders.

Case Study 2. Pressure on reindeer pastures in the Yamalo-Nenets Autonomous District

Area: Yamalo-Nenets Autonomous District (Fig. S2)

Latitude: 66 – 73 °N

Population: out of 0.5 million people in the Region, around 30 000 are Nenets.

Environment: tundra and forest-tundra,

Research Station/authority: Arctic Research Center of the Yamalo-Nenets Autonomous District (Fig. S2).

Key stakeholders:

- collective reindeer herding enterprises (called “sovhoz”)
- individual reindeer herders and their families
- local government Department of Indigenous Peoples
- local government Department of Agriculture
- oil and gas industry (GAZPROM, NOVATEK, Total, etc.)

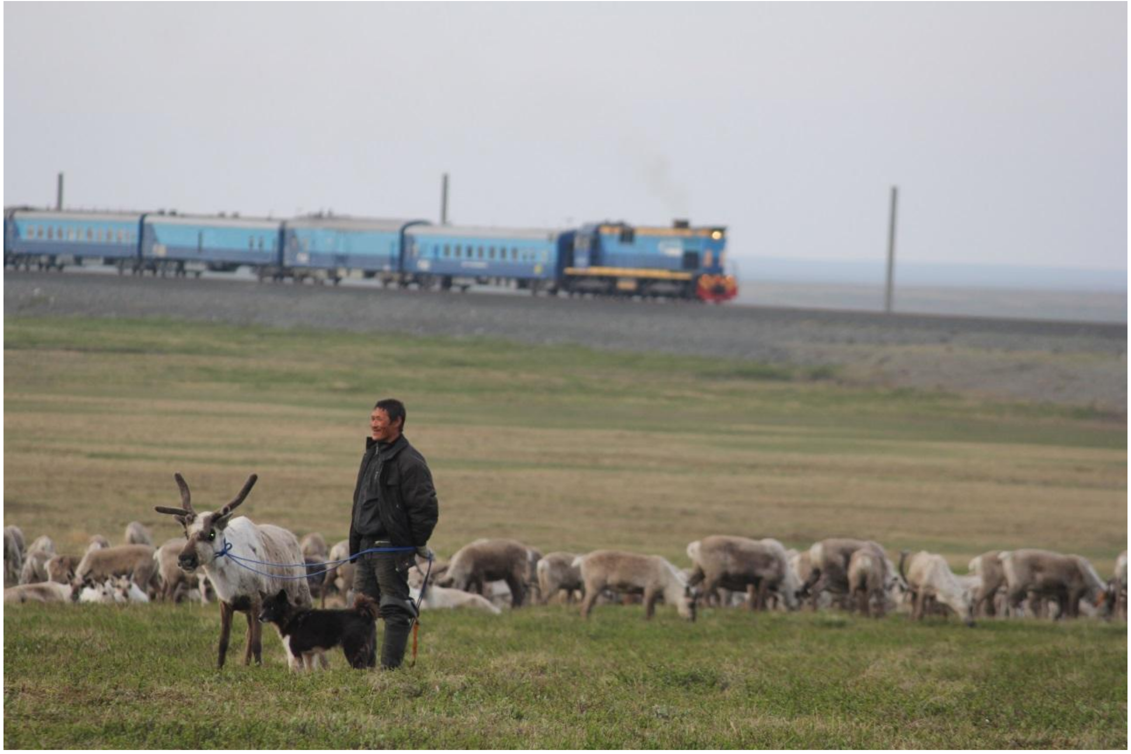

Fig. S2. Reindeer herding and modern development. Nenets Reindeer herders in the tundra of Yamal. Photo: Marina Yar

Background

There is a current problem of reindeer pasture deterioration in the Yamalo-Nenets Autonomous District. After the collectivization in the middle of the 20th century, the reindeer husbandry started to transform from the traditional way of life of Nenets People to a meat and antlers producing industry. Though Nenets preserved the nomadic lifestyle, the introduction of vaccination of the animals led to uncontrolled growth of the reindeer population, that doubled from the 1960s to 2000 and has continued to expand rapidly from then (Bogdanov and Golovatin 2017). In addition, the reindeer pastures have been reduced and fragmented by industrial development of the oil and gas industry (Kumpula et al. 2011) and encroachment of shrubs into the tundra (Frost et al. 2018). On the other hand, we argue that the impact of industrial development is minor in comparison with the overgrazing. According to our satellite image analysis (Anonymous, *in prep*), during the last 10 years the amount of land deterioration by construction works, roads and pipelines increased a lot (from 0,01% to 0,2% of the total land area) but heavily overgrazed tundra currently occupies more than 30% of the territory. Legislation to reduce reindeer herd numbers does not exist in Russia yet, neither on regional nor federal levels.

Aim

The aim of this case study is to explore the complexities and impacts of overgrazing by reindeer and the roles of local peoples, researchers and decision makers in resolving the issues so as to sustain both traditional livelihoods and reindeer pastures.

Existing process

Societal aspects contributing to the problem are mainly the following 1) Around half of the herds belongs to private households and as the culture of the Nenets regards success in terms of large numbers of reindeer, the families would rather keep the calves than get monetary profit from selling the meat. 2) The collective reindeer herding enterprises (sovhoz) get subsidies for each reindeer they have, so each tries to graze more animals than the others. 3) It is a lot more efficient to sell reindeer antlers to illegal resellers and keep the herd numbers, than reduce it every year by selling meat (the other problem is that the antlers market is totally uncontrolled by the Government).

In 2016, an outbreak of anthrax on the Yamal Peninsula killed two people, several thousand reindeer and drew the attention of the regional government to the problem (Bogdanov and Golovatin 2017). The problem had arisen because uninfected land available for grazing was overgrazed and could not sustain the current reindeer stocks any more. Consequently, herders started to use the infected lands that had been excluded previously from the reindeer pastures.

To stabilize the situation and prevent a major disaster for people and reindeer, decision makers (the regional government) must act but several intermediate objectives have to be reached:

- The data on the exact number of reindeer should be available for the decision makers. However, this task is more complex, than it seems at first sight. The tradition of Nenets reindeer herders considers it impolite to ask about the size of the heard directly (the herd can be considered as a "bank account"!), so when the authorities order them to report many do

not tell the truth, mostly lowering the numbers. On the other hand, the “sovhozes” are interested in overstating the numbers of reindeer in their possession. Currently the total number of reindeer in the Yamalo-Nenets Autonomous District is estimated as 700 000±200 000. However, a mechanism for collecting verifiable numbers has not been developed yet.

- The current reindeer capacity of the pastures should be calculated from a geobotanical survey. However, the state of tundra and taiga vegetation throughout the region was last mapped in the late 1970s and since then only local studies by diverse research groups have been done without assessing the state of the area’s vegetation as a whole. In 2017, a survey was started in the most crucial tundra regions of the Yamalo-Nenets Autonomous District, i.e. the Yamalskiy and Tasovskiy Districts. All the scattered geobotanical data from the Region (mostly unpublished) collected in the last 10 years was used to identify the most under-studied sites. In the summer of 2017, three groups of researchers from several institutions from all around Russia conducted vegetation surveys and the following detailed maps were created: 1) vegetation, 2) total phytomass and 3) summer and winter reindeer capacities. According to this study the winter capacity of tundra pastures is now exceeded by more than 20 times. In the future, it is planned to map the southern part of the region in the same way.

Recommendations

There should be legal regulation of reindeer numbers and the regulations should be based on the status of the land (according to the reindeer capacity) and of the reindeer herders being ether just people leading a traditional way of life, or farmers getting profit from selling meat and antlers (according to the number of animals they herd). The regulations should determine the rights of

each family/enterprise to use prescribed land allotments. However, we should consider possible obstacles in getting to a consensus with the local indigenous communities: most of the Nenets are against any interference of the Government in their herding strategy and any attempts of regulating it would lead to strong conflicts between herders of different status. In Norway, an attempt to regulate the number of reindeer herders by forcing out the herders with low numbers of reindeer resulted in severe social problems among many of those that were forced out of reindeer herding, despite sufficient monetary support that these people received (Bergland 2005); https://lovdata.no/dokument/JB/forskrift/2003-12-19-1858).

The first obvious problem the authorities face is that it is impossible to calculate the exact numbers of reindeer each family has, and the herders are strongly against the introduction of ear tagging of all the domesticated animals, that is now obligatory throughout Russia.

The search for the solution of the problem has just started but it is already obvious that the only way to get progress is through collaboration among:

- scientists, producing actual data of the state of the pastures and the animals,
- local and federal authorities providing essential regulation and legislation and
- the Nenets herders, helping to frame appropriate and fair legislation but with the goals of sustaining reindeer husbandry in a way that would save both the traditional way of life and the pastures.

The first dialog between the Nenets and scientists occurred in the center of the Yamalskiy District settlement at Yar-Sale on 7th April and in the Tazovskiy District on 17th of May of 2018 where the heads of indigenous communities agreed on the scale and the acuteness of the problem and an urgent need of action to solve it.

Case Study 3. Reindeer herding and tourism in Scandinavia

Area: Northern Scandinavia in the sub-Arctic (Fig. S3)

Latitude: 63.44 – 71.17°N

Population: 1.5 million of which 13,000 are Sámi connected to reindeer herding (Norway Sweden, Finland)

Environment: Arctic tundra, sub-Arctic birch forest tundra, boreal forest.

Research stations: Abisko, Tarfala, Kiplisjarvi, Kevo, Bioforsk Svanhovd

Key stakeholders:

- Reindeer herders
- Herding authorities
- Research institutions (Stations and Universities including Tromsø, and Umeå)
- Forest industry
- Mining industry
- Hunting and fishing associations
- Conservation authorities
- Tourism businesses

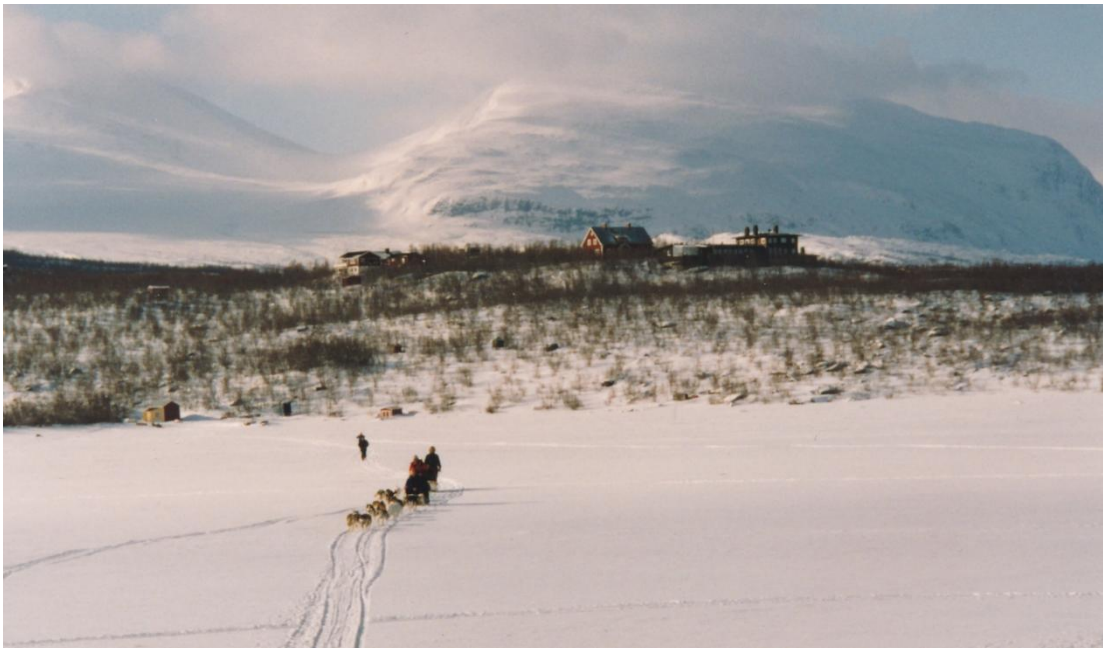

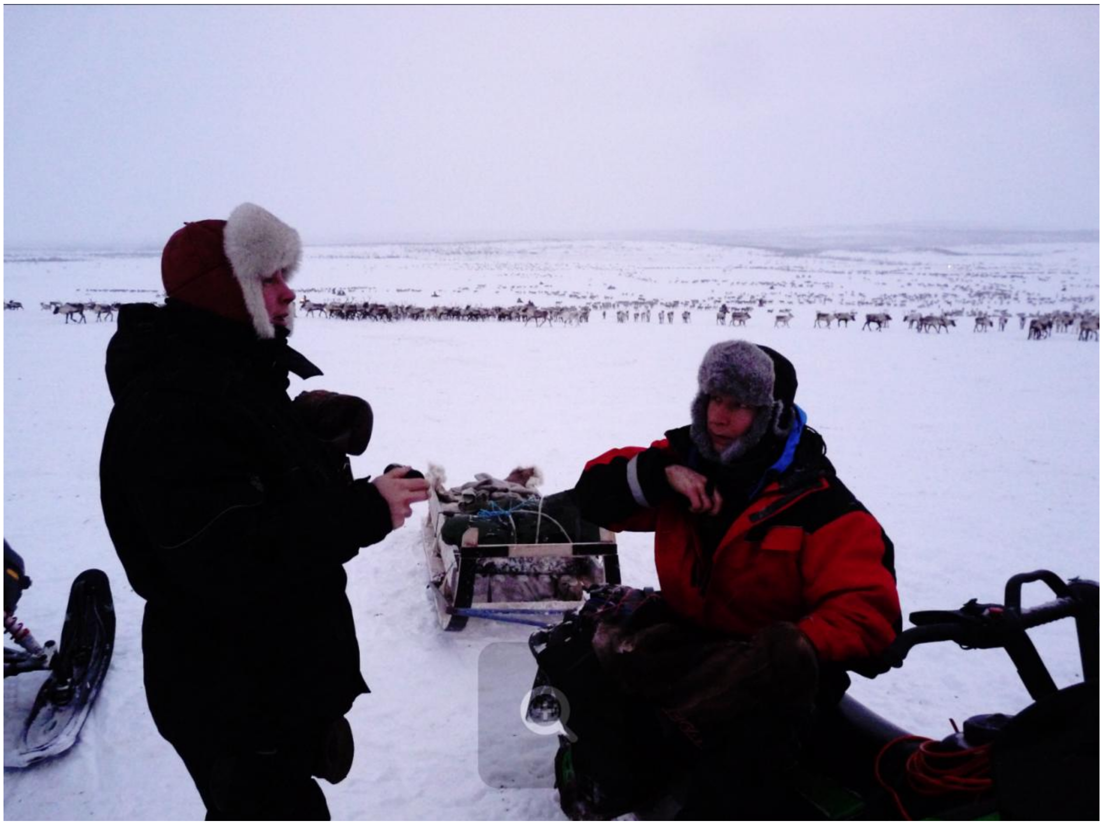

Fig. S3. Tourist dog sledging – above on Lake Torneträsk near the Abisko Research Station in Swedish Lapland. Photo: T.V. Callaghan, below in reindeer herds. Photo: N. Labba.

Background

In central Europe, tourism businesses depending on the snow season are experiencing shorter seasons due to the warmer climate and they are looking to relocate some activities to Scandinavia. In Scandinavia, climate change is creating environmental and social transformations that have a combined, if not amplified, effect on reindeer herding and traditional livelihoods.

Environmental changes are negatively affecting the production capacity of the reindeer due to the higher frequency of thawing and icing events (Johansson et al. 2011). These events create ice layers that damage vegetation (Bokhorst et al. 2009; Eira et al. 2018) and prevent reindeer from reaching available food, with the consequences of starvation and a high rate of mortality. This has a negative effect on the reindeer herder's economy.

The social transformations resulting from climate change include greater human activity in reindeer herding areas, where tourism is the fastest growing activity. Reindeer are disturbed by tourism activities such as dog-sledging (Fig. S3), and in some cases, local areas can no longer be used as winter grazing pastures. Although some reindeer herders have adapted and are benefitting from the new economic opportunity that tourism has created, the majority of the herders are experiencing a threat from the increasing tourism.

Aim

To explore the combined impacts of increased tourism and environmental change on reindeer herders and to discuss possible resolutions.

Existing process

Organizations coordinating tourism in the Scandinavian countries report that there is a growth in the number of companies offering different types of winter activities (Committee on Regional Development, 2013). Short trips with sledge dogs to see the *aurora borealis* have the largest growth, but there is also an increase in longer trips in the wilderness. These longer wilderness trips have their origin in Canadian sledge dog races. International companies selling outdoor equipment and winter clothing use extreme sports such as sledge dog races as marketing. This marketing reaches a large public and creates a demand for sledge dog wilderness tours, and the same companies are sponsoring some larger sledge dog races.

The “Finnmarkløpet” in northern Norway is Europe´s longest dog sledge race with a track of 1200 km through reindeer herding winter grazing areas. Despite this, there are no reports of disturbances on reindeer herding. The main reason for this is that the organizers have a good dialogue with the local reindeer herders. The Finnmarksløpet is a good example of how it is possible to minimize or eliminate disturbances from sledge dogs on reindeer herding through planning and dialogue. This race could be a good opportunity for reindeer herders and tour operators to share information about their own businesses and their demands, and to develop some form of mutual understanding without the need for new legislation.

Some sledge dog operators are starting to market wilderness tours in Scandinavia using the liberal Scandinavian “nature use law” that legislates free travel. These “Allemannsrätten” (Everyone’s Rights) give all citizens, domestic or visitors, the right for free travel without motorized vehicles in all areas, and such access to nature reserves is not restricted. On the other hand, reindeer herders do not own the land that they use: the land is mainly state owned but Sámi reindeer herding has an immemorial right (Rennäringslagen, 1971)

Reindeer herders meet European sledge dog operators while they are conducting their wilderness tours. Some European operators have little or no knowledge about reindeer herding and they do not know what problems they cause for this long-established and former essential survival activity. During wintertime, reindeer need to save energy by being stationary in one grazing area. If the tour operators do not respect this, and instead search for reindeer to show to the tourists, or set up temporary camps close to the herds, they will cause disturbance. Such disturbance will have an even more harmful impact when climate change with its thawing and freezing events creates starvation conditions that weaken the reindeer. During springtime, reindeer migrate towards the summer grazing land. If the dog sledge operators do not know these routes, or do not respect them, there will also be disturbance in this season too as reindeer will be forced to choose other routes and will end up in the wrong summer grazing areas, with more work for the reindeer herders.

Recommendations

The successful dialogue between reindeer herders and the operators of the Finnmarkløpet can be used as a model for other tourist activities and has the advantage that changes to legislation by decision makers will be avoided and the important and exemplary Allemannsrätten can be maintained. Measures such as the Finnmarksløpet strengthen the access to, and increase the interest for nature, and the majority of society will then support the needs of the reindeer herders. This will make the reindeer herders' voice more important, especially in discussions about concessions for extractive industries.

In cases where the Allemannsrätten are abused to favour activities by foreign businesses, decision makers such as local and national governments should sadly consider new and appropriate legislation to maintain traditional livelihoods, landscapes, flora and fauna while

allowing harmless business initiatives. The supreme court of Sweden has made a judgment that forbids business activities on another man's private land if it has a negative economic effect for the landowner (NJA 1996 s 495 (https://lagen.nu/dom/nja/1996s495)). For reindeer herders, this supreme court decision does not change the situation: the herders do not own the land that they use for reindeer herding, and it is difficult for the herder to verify the negative economic effects that are directly connected to tourism.

Although this case study focuses on current business trends and environmental change, it is likely that impacts of future climate change such as winter heat stress, snow cover reduction, shrub and tree expansion etc. will impose even greater stresses on reindeer herding. Consequently, there is a need for forward-looking activities in which researchers predict future environmental conditions and develop optimisation plans for multiple land uses while sustaining and prioritising traditional livelihoods.

REFERENCES

Anonymous In Prep. Geobotanical Assessment of the Yamal-Nenets Autonomous District

Bergland, E. 2005. Reindrift, Omstilling og identitet (Reindeer herding, restructuring and identity). *Dieđut* 1: 210.

Bogdanov, V. D., and M. G. Golovatin. 2017. Anthrax in Yamal: An ecological view on traditional reindeer husbandry. *Russian Journal of Ecology* 48: 95–100. doi:10.1134/S1067413617020059.

Bokhorst, S. F., J. W. Bjerke, H. Tømmervik, T. V. Callaghan, and G. K. Phoenix. 2009. Winter warming events damage sub-Arctic vegetation: consistent evidence from an experimental manipulation and a natural event. *Journal of Ecology* 97. John Wiley & Sons, Ltd (10.1111): 1408–1415. doi:10.1111/j.1365-2745.2009.01554.x.

Committee on Regional Development, Report of the Delegation to Kiruna Sweden 16-18 June 2013

Eira, I. M. G., A. Oskal, I. Hanssen-Bauer, and S. D. Mathiesen. 2018. Snow cover and the loss of traditional indigenous knowledge. *Nature Climate Change* 8. Nature Publishing Group: 928–931. doi:10.1038/s41558-018-0319-2.

Federal Law "On Recreational Fishing" # 475, 25.12.2018 (in Russian)

http://www.consultant.ru/document/cons_doc_LAW_314261/

Frost, G. V., H. E. Epstein, D. A. Walker, G. Matyshak, and K. Ermokhina. 2018. Seasonal and Long-Term Changes to Active-Layer Temperatures after Tall Shrubland Expansion and Succession in Arctic Tundra. *Ecosystems* 21. Springer US: 507–520. doi:10.1007/s10021-017-0165-5.

Halleyl, D. J., and F. Roself. 2003. Population and distribution of European beavers (*Castor fiber*). *Lutra* 46: 91– 101. doi:10.4067/S0717-92002008000200009.

Johansson, C., V. A. Pohjola, C. Jonasson, and T. V. Callaghan. 2011. Multi-Decadal Changes in Snow Characteristics in Sub-Arctic Sweden. *AMBIO* 40. Springer Netherlands: 566–574. doi:10.1007/s13280-011-0164-2.

Kumpula, T., A. Pajunen, E. Kaarlejärvi, B. C. Forbes, and F. Stammler. 2011. Land use and land cover change in Arctic Russia: Ecological and social implications of industrial development. *Global Environmental Change* 21: 550–562. doi:10.1016/j.gloenvcha.2010.12.010.

Popkov, V. K., L. A. Popkova, and A. I. Ruzanova. 2008. Ecological characteristics of the *Abramis brama* (L.) and the consequences of its acclimatization in the basin of the Middle Ob river. *Bulletin of Tomsk State University*: 154–157.

Popkov, V. K., V. V. Drozdov, and O. G. Nekhoroshev. 2018. Preliminary data to assess the impact of beaver dams on fish migration and the formation of fish resources in the Middle Ob River flood plain (Tomsk Region) *IOP Conf. Series: Earth and Environmental Science* 201 (2018) 012016 doi :10.1088/1755-1315/201/1/012016

Puttock, A., H. A. Graham, A. M. Cunliffe, M. Elliott, and R. E. Brazier. 2017. Eurasian beaver activity increases water storage, attenuates flow and mitigates diffuse pollution from intensively-managed grasslands. *Science of The Total Environment* 576. Elsevier: 430–443. doi:10.1016/J.SCITOTENV.2016.10.122.

Rakhmanova, L. 2018. Trial – adaptation - rooting: understanding and embodiment of the concepts in the Arctic and Siberia. In *Paths of Russia. 1917-2017: one hundred years of changes*, 227–244. Moscow, St. Petersburg: Nestor-History.

Rennäringslagen, 1971:437

Russian Census for 2010
https://tmsk.gks.ru/wps/wcm/connect/rosstat_ts/tmsk/ru/census_and_researching/census/national_census_2010/score_2010/
